# Developmental regulation of leaf venation patterns: monocot versus eudicots and the role of auxin

**DOI:** 10.1111/nph.17955

**Published:** 2022-02-02

**Authors:** Chiara Perico, Sovanna Tan, Jane A. Langdale

**Affiliations:** ^1^ Department of Plant Sciences University of Oxford South Parks Rd Oxford OX1 3RB UK

**Keywords:** auxin, eudicots, leaf development, leaves, monocots, venation patterning

## Abstract

Organisation and patterning of the vascular network in land plants varies in different taxonomic, developmental and environmental contexts. In leaves, the degree of vascular strand connectivity influences both light and CO_2_ harvesting capabilities as well as hydraulic capacity. As such, developmental mechanisms that regulate leaf venation patterning have a direct impact on physiological performance. Development of the leaf venation network requires the specification of procambial cells within the ground meristem of the primordium and subsequent proliferation and differentiation of the procambial lineage to form vascular strands. An understanding of how diverse venation patterns are manifest therefore requires mechanistic insight into how procambium is dynamically specified in a growing leaf. A role for auxin in this process was identified many years ago, but questions remain. In this review we first provide an overview of the diverse venation patterns that exist in land plants, providing an evolutionary perspective. We then focus on the developmental regulation of leaf venation patterns in angiosperms, comparing patterning in eudicots and monocots, and the role of auxin in each case. Although common themes emerge, we conclude that the developmental mechanisms elucidated in eudicots are unlikely to fully explain how parallel venation patterns in monocot leaves are elaborated.

1

**Table:** 

	Contents	
	Summary	783
I.	Introduction	783
II.	Leaf venation patterns in angiosperms	785
III.	Auxin – synthesis, transport and signalling	786
IV.	Ontogeny of leaf veins	790
V.	Future perspectives	798
	Acknowledgements	798
	References	798

## I. Introduction

**Fig. 1:**
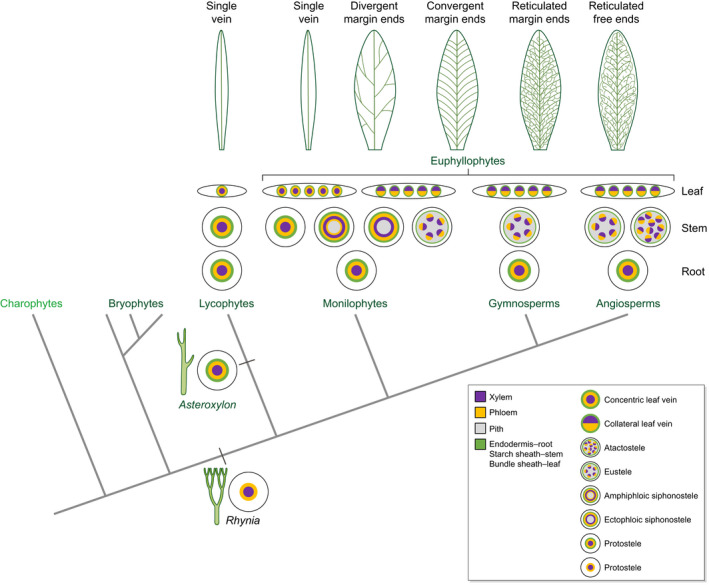
Vascular patterning in land plants. Land plants evolved from charophyte algae *c*. 470 million years ago (Ma) and vasculature evolved *c*. 430 Ma (Kenrick & Crane, 1997). Stellar patterns in radial axes of extinct (black) and extant (green) plant groups are indicated. Protostele is observed in the stems of some extant lycophytes and ferns (albeit with structural variations in terms of the geometry of the xylem), and in the roots of all vascular plants (Beck *et al*., 1982; Tomescu, 2021). For simplicity, subtypes of protostele that display different xylem geometries are not included. Derived arrangements that include pith tissue are seen in the stems of most ferns (siphonostele), *Equisetum* (eustele) and all seed plants (eustele or atactostele), but their respective evolutionary trajectories from protostele are not clear. A sheath layer is absent in *Rhynia* (Kidston & Lang, 1917) but was reported in *Asteroxylon* (Kidston & Lang, 1920). Leaf venation patterns are represented in transverse and paradermal views. In extant species, xylem and phloem arrangements are concentric in lycophyte leaves but collateral (polarised in the adaxial–abaxial leaf axis) in seed plants, as indicated. Both concentric and collateral forms are seen in extant monilophytes (Parihar, 1966). Along the apical–basal leaf axis, single veins are a feature of extinct and extant lycophytes, of some extant monilophytes following reversion, and of some extant gymnosperms. Divergent, convergent and reticulated patterns are represented in the evolutionary trajectory of monilophytes, gymnosperms and angiosperms, but not all are seen in extant taxa of each group (Boyce & Knoll, 2002).

The evolution of vasculature in land plants released developmental constraints on plant architecture by providing both mechanical support and conduits to transport water and solutes between above‐ground and below‐ground structures. Although water‐conducting vessels and food‐conducting cells are found in nonvascular plants (bryophytes), they lack lignin and therefore have a limited role in mechanical support or hydraulics (Ligrone *et al*., 2012). In the earliest vascular plants (e.g. extinct *Cooksonia* and *Rhynia*) the vasculature of the main axis were comprised of a core of xylem surrounded by a ring of phloem (Kidston & Lang, 1917, 1920). This ancestral ‘protostele’ was modified during the course of land‐plant evolution but the root and stem vasculature was always radially patterned in the context of a cylindrical growth axis (Fig. 1).

Extant vascular plants (tracheophytes) in lycophyte, monilophyte (ferns and horsetails) and seed plant (gymnosperms and angiosperms) lineages all have leaves of some form; however, leafless fossils suggest that extensive branching of vascularised growth axes occurred before leaf evolution (Kenrick & Crane, 1997). These same fossils revealed that leaves evolved independently on multiple occasions (Boyce & Knoll, 2002; Harrison & Morris, 2018). Although the exact number of events continues to be debated, there is general consensus that lycophyte leaves evolved independently from those of euphyllophytes (monilophytes and seed plants), and that at least two independent events occurred within the euphyllophytes, with one in seed plants and the remainder in monilophytes (Tomescu, 2009). On each occasion that leaves evolved, at least three developmental mechanisms had to be modified: the first to enable the transition from indeterminacy in the main growth axis to determinacy in a lateral organ; the second to developmentally pattern planar, flattened structures; and the third to connect tissues of the lateral organs to the vasculature of the main axis. Comparative analyses between the lycophyte *Selaginella kraussiana* and the model angiosperm *Arabidopsis thaliana* (Arabidopsis) suggest that the same developmental mechanism was recruited in parallel during the evolution of determinate lateral organs in lycophytes and seed plants (Harrison *et al*., 2005). In both cases, KNOTTED1‐like (KNOX) homeodomain transcription factors promote indeterminacy in the shoot, and determinacy in the leaf is maintained through suppression of KNOX activity by MYB‐like ARP transcription factors (Harrison *et al*., 2005). By contrast, expression patterns of genes encoding Class III HD‐ZIP transcription factors in extant lycophytes, ferns and seed plants suggest that the establishment of organ polarity and the elaboration of distinct venation systems in lycophyte and euphyllophyte leaves could have been mediated by different developmental mechanisms in the two lineages (Floyd & Bowman, 2006; Prigge & Clarke, 2006; Vasco *et al*., 2016; Zumajo‐Cardona *et al*., 2019). This suggestion is consistent with the proposal that leaf venation systems evolved in different contexts, either in nonvascularised lateral outgrowths (Bower, 1908; Kenrick & Crane, 1997) or through modifications of branched stems (Zimmermann, 1952).

The evolution of leaves increased the capacity for photosynthesis by expanding the surface area available to capture light and CO_2_, however, the extent to which that capability was realised was linked to the capacity of the vascular system to move water into the leaf (de Boer *et al*., 2012; Sack & Scoffoni, 2013). Fossil evidence suggests that early leaves of all vascular plant groups were small structures with a single vein per lamina segment (Boyce & Knoll, 2002). Given that hydraulic modelling has demonstrated how single veins can constrain both the size and shape of the leaf that they serve (Zwieniecki *et al*., 2004), it is therefore likely that the evolution of diverse leaf morphologies was intrinsically linked to the development of increasingly complex venation systems. Indeed, the interdependency of venation complexity and leaf growth has been proposed to explain the diversity of leaf shapes (Runions *et al*., 2017). Such a link, particularly in relation to shape variations such as serrations, is proposed to have functional significance in terms of influencing both water conductance and photosynthetic capacities (Brodribb *et al*., 2010; Nicotra *et al*., 2011). Notably, increases in venation complexity followed the same trajectory in monilophyte and seed plant lineages, with divergent, convergent and reticulate patterns of veins that ended at the leaf margins emerging sequentially (Boyce & Knoll, 2002; Rothwell *et al*., 2014) (Fig. 1). The final pattern to emerge, reticulate venation with veins ending internally in the lamina, is generally associated with leaves of dicotyledonous angiosperms, but examples can be found in extant ferns (e.g. polypods and dryopterids) and gymnosperms (e.g. Gnetales). Reversion to single vein leaf forms has been documented, with multiple examples in monilophytes (Kenrick & Crane, 1997; Corvez *et al*., 2012), but in all cases this reversion was accompanied by a reduction in leaf size. In combination, evidence from both extinct and extant plant groups suggests that developmental mechanisms that elaborate leaf venation patterns both regulate and are regulated by organ growth controls.

This review draws together historical and recent research that sheds light on how leaf venation patterns are regulated in extant land plants. Whilst recognizing the range of patterns that are evident across lineages, the main focus is on angiosperms, and specifically on a comparison between developmental mechanisms in eudicotyledonous (eudicot) and monocotyledonous (monocot) species. Although the leaf venation system in any plant is an integral part of the shoot–root vascular network, patterning and cell‐type differentiation in the embryo, root and stem vasculature is not discussed here because the topics have been extensively covered elsewhere (e.g. Lucas *et al*., 2013; De Rybel *et al*., 2016; Hellmann *et al*., 2018; Fischer *et al*., 2019; Fukuda & Hardtke, 2020; Ohashi‐Ito & Fukuda, 2020).

## II. Leaf venation patterns in angiosperms

**Fig. 2:**
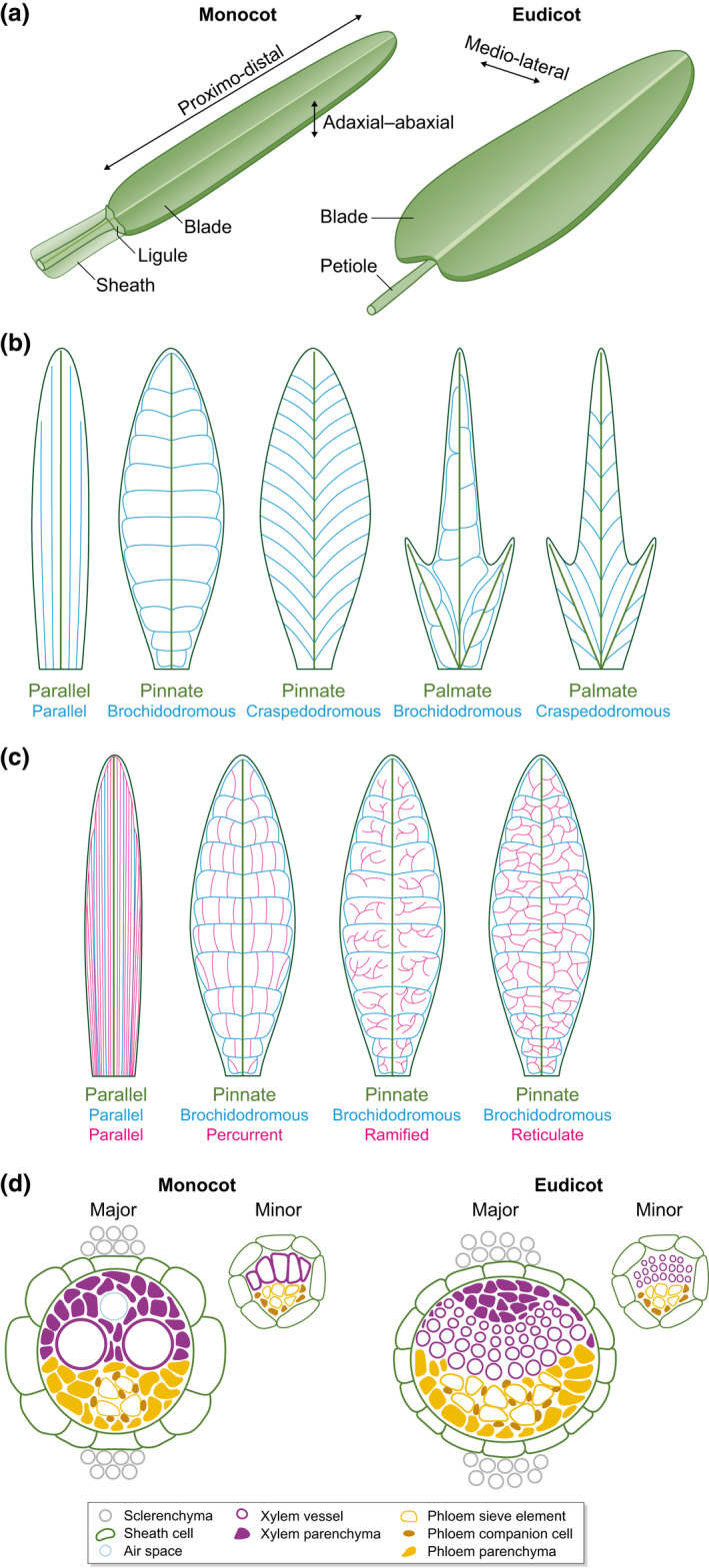
Leaf venation in angiosperms. (a) Schematic showing axes and domains in simple leaves of monocots and eudicots. The midvein, which extends from the base of the sheath to the tip of the blade (monocots) or from the petiole to the tip of the blade (eudicots), is indicated. In grass leaves the blade and sheath regions are demarcated by a ligule. (b) Patterns of primary (green) and secondary (turquoise) veins observed in monocot (parallel only) and simple eudicot leaves. (c) Patterns of tertiary (pink) veins observed in monocot leaves (parallel only) and in simple eudicot leaves with a pinnate brochidodromous scaffold. The three different tertiary patterns in eudicots can also be found in palmate brochidodromous, pinnate craspedodromous and palmate craspedodromous leaves. (d) Schematic transverse sections of leaf veins in monocots and eudicots. The adaxial side is uppermost.

With few exceptions, leaf venation patterns in angiosperms are elaborated in a flattened organ, with patterning occurring as proximo‐distal (base to tip), medio‐lateral (midrib to margins) and adaxial–abaxial (upper to lower) axes (Fig. 2a) are being established, as well as during later stages of growth (reviewed in Satterlee & Scanlon, 2019). In monocots, the leaf commonly comprises a distal blade and a proximal sheath, whereas in eudicots the blade is subtended by a petiole. In both cases, vein networks develop hierarchically with lower order (major) veins developing before higher order (minor) veins (Esau, 1965; Nelson & Dengler, 1997). This hierarchy, which is both spatially and temporally regulated by the dynamic interplay between organ growth, axis formation and vascular differentiation, yields network topologies that are classified on the basis of the number, position and connectivity of veins (Hickey, 1973; Roth‐Nebelsick *et al*., 2001; Sack & Scoffoni, 2013) (Fig. 2b,c).

In most monocot leaves, the major vein ranks align in parallel along the proximo‐distal axis, with the primary (composite midvein), secondary (laterals) and tertiary (‘rank 1’ intermediates) veins forming in sequence. All of these vein ranks are classified as ‘major’ in that the ring of sheath cells encircling the vein is capped by sclerenchyma (bundle sheath extension cells) on the adaxial and/or abaxial side (Esau, 1965) (Fig. 2d). Minor vein ranks include the nonsclerified transverse veins that develop parallel to the medio‐lateral axis to create a closed grid plus the longitudinal ‘rank 2 intermediate’ veins that generate the high vein densities typical of C_4_ grass leaves (Ueno *et al*., 2006; Sedelnikova *et al*., 2018). Although examples of nonparallel systems can be found in monocots (e.g. banana) the otherwise uniformity of venation patterns is consistent with the relatively invariant strap shape of many monocot leaves (particularly in the grasses), but is in sharp contrast with the diverse range of patterns and shapes in eudicot leaves. In both simple and compound leaves of eudicots, the primary veins comprise either the single midvein extending along the proximo‐distal axis (pinnate) or the midvein plus a small number of veins branching from it towards the margins (palmate) (Fig. 2b). In most cases the secondary veins emerge from the pinnate or palmate scaffold to form either closed loops (brochidodromous) (Fig. 2b), an array of single veins that terminate at the leaf margin (craspedodromous) (Fig. 2b) or a combination of the two. Finally, tertiary veins form percurrent, ramified or reticulate patterns (Fig. 2c), yielding either open or closed networks depending on the precise combination of primary, secondary and tertiary vein structures. In general terms, therefore, leaf venation patterns in monocots tend to be parallel and closed whereas those in eudicots are reticulate and can be either open or closed.

In addition to recognizable topologies in the proximo‐distal and medio‐lateral axes, leaf veins develop with adaxial–abaxial polarity. In the majority of angiosperms, xylem is positioned adaxially and phloem abaxially (Fig. 2d). Within the xylem and phloem tissue, cell‐type differentiation varies between major and minor veins. For example, major veins have higher proportions of large metaxylem vessels than minor veins. Different proportions of phloem cell types are also found because major veins generally unload sugars in sink leaves (while minor veins are still developing), whereas minor veins upload sugars in source leaves (Haritatos *et al*., 2000; Rennie & Turgeon, 2009). Although the specifics of xylem and phloem differentiation will not be considered further (reviewed in Schuetz *et al*., 2012; Sack & Scoffoni, 2013; Furuta *et al*., 2014; Anne & Hardtke, 2018; López‐Salmerón *et al*., 2019), adaxial–abaxial patterning is discussed in more detail in a subsequent section (Section IV).

The leaf venation patterns discussed previously have long intrigued both natural and physical scientists. Now, more than 50 years after Sachs first proposed that the phytohormone auxin induced the differentiation of vascular tissue after wounding (Sachs, 1969), there is still much to learn about how auxin homeostasis influences (and is influenced by) vein formation. The rest of the review focuses on how auxin synthesis, transport and signalling pathways interact with both genetic and biophysical parameters to pattern the leaf venation network, with an emphasis on mechanistic differences between monocots and eudicots.

## III. Auxin – synthesis, transport and signalling

Auxin regulates numerous developmental processes (reviewed in Scarpella *et al*., 2010; Zhao, 2018; Swarup & Bhosale, 2019) and therefore its activity has to be tightly regulated both spatially and temporally. Such regulation requires finely tuned coordination between *de novo* synthesis, storage, degradation, transport and signalling, each of which is discussed in later sections (Sections III..1, III..2, III..3, III..4). We also discuss the potential and limitations of methods that have been used to detect auxin activity *in vivo* during leaf development (Section III.5). Finally, we speculate on how the evolution of auxin pathway components may have facilitated the development of vascular tissues (Section III.6).

### III..1 Synthesis

**Fig. 3:**
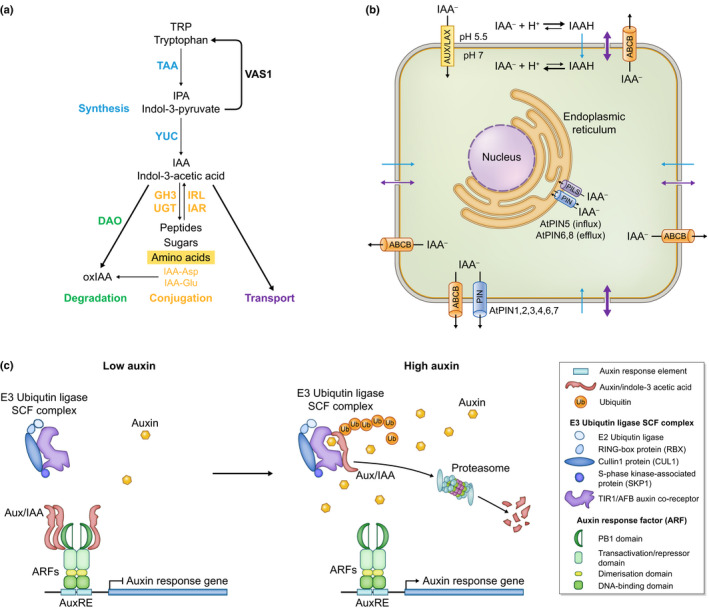
Overview of auxin metabolism, transport and signalling. (a) Auxin levels are maintained through *de novo* synthesis (blue), conjugation (yellow) and degradation (green). In the well characterised TAA/YUC tryptophan (Trp)‐dependent synthesis pathway, Trp is initially converted into indol‐3‐pyruvate (IPA) by tryptophan aminotransferase (TAA) enzymes. IPA is then converted into indol‐3‐acetic acid (IAA), which is the most common form of auxin, by YUCCA (YUC) enzymes. The levels of IPA intermediate available in a cell can be controlled by the pyridoxal phosphate‐dependent aminotransferase VAS1, which converts IPA back into Trp. Conjugation with amino acids, peptides or sugars regulates the amount of biologically active auxin with enzymes such as Gretchen Hagen 3 (GH3) and UDP‐glucosyl transferase (UGT) conjugating IAA with amino acids or sugars, respectively. Conjugation with amino acids such as alanine and leucine is generally reversible via enzymes such as IAA‐LEUCINE RESISTANT 1‐like (IRL1) and its homologue IAA‐ALANINE RESISTANT 3 (IAR3), whereas conjugation with aspartic acid (Asp) or glutamic acid (Glu) flags auxin for oxidation to 2‐oxindole‐3‐acetic acid (oxIAA) and subsequent degradation. oxIAA can also be generated independently of conjugation by the enzyme DIOXYGENASE FOR AUXIN OXIDATION (DAO). (b) Summary of auxin transport pathways identified in *Arabidopsis thaliana*. Auxin can move in and out of the cell via plasmodesmata (purple arrow) and diffuse in as IAAH (blue arrow). Other flux is mediated by transporter proteins that are localised in the plasma membrane or endoplasmic reticulum. AUX/LAX (yellow) transporters move auxin into the cell whereas PIN (blue) and ABCB (orange) transporters move auxin out of the cell. Transport between the cytoplasm and endoplasmic reticulum is mediated by PIN (influx and efflux) and PIN‐LIKES (PILS; grey) (influx only) transporters. (c) Canonical auxin signalling pathway. Auxin response factor (ARF) transcription factors bind to auxin response elements (AuxRE) in the promoters of auxin responsive genes. At low concentrations of auxin, Aux/IAA proteins interact with ARFs to inhibit their activity whereas at high concentrations auxin mediates an interaction between Aux/IAA and the TIR1/AFB F‐box protein of the E3 Ubiquitin ligase Skp, Cullin, F‐box containing (SCF) complex. This interaction leads to polyubiquitination of the Aux/IAA and subsequent degradation by the proteasome. As a consequence, ARF inhibition is relieved enabling expression or repression of the auxin responsive gene depending on whether the ARF is an activator or a repressor (only activation is shown).

The most abundant auxin molecule, indole‐3‐acetic acid (IAA), can be synthesised *de novo* in plants in a tryptophan (Trp)‐dependent or Trp‐independent way. The existence of a Trp‐independent pathway was postulated because IAA was detected in Trp auxotroph mutants (Wright *et al*., 1991; Normanly *et al*., 1993), but details of the pathway are scarce. By contrast, four different Trp‐dependent pathways have been identified (reviewed in Zhao, 2012; Kasahara, 2016; Zhao, 2018). The best characterised is the two step tryptophan aminotransferase (TAA)/YUCCA (YUC) pathway in which TAA first removes the amino group from Trp to form indole‐3‐pyruvate (IPA) and then IPA is decarboxylated by YUC to produce IAA (Zhao *et al*., 2001; Stepanova *et al*., 2011; Won *et al*., 2011) (Fig. 3a, blue). Despite the apparent simplicity of this two‐step pathway, it was initially difficult to characterise because of the number of homologous genes involved. For example, 4 *TAA* and 11 *YUC* genes are found in Arabidopsis (Zhao *et al*., 2001; Cheng *et al*., 2006; Stepanova *et al*., 2008, 2011). However, IAA synthesis can be disrupted through mutation of multiple paralogues or by pharmacological inhibition and in each case decreased IAA is associated with the development of a disorganised and fragmented venation network (Stepanova *et al*., 2008, 2011; Nishimura *et al*., 2014), suggesting that auxin biosynthesis is involved in regulating leaf venation patterns.

### III..2 Storage and degradation

Some mechanisms operate to ensure that bioactive auxin accumulates at the right level in the appropriate spatial and temporal contexts throughout development. The first (Fig. 3a, blue) is enabled by the complexity of the *YUC* and *TAA* gene families, which allows for context‐dependent accumulation of different YUC and TAA enzymes, and therefore for tissue‐ and/or stage‐specific regulation of auxin synthesis (Cheng *et al*., 2006). The second controls the amount of auxin synthesised by regulating levels of the YUC/TAA pathway intermediate IPA. In this case, the aminotransferase VAS1 (Fig. 3a) acts as a rheostat to coordinately regulate intermediates of the auxin and ethylene biosynthesis pathways, converting IPA back to Trp when levels are too high (Zheng *et al*., 2013). The third control system (Fig. 3a, yellow) regulates the amount of active auxin that is locally available (reviewed in Ludwig‐Muller, 2011). Free IAA is the active form of auxin whereas IAA conjugated with sugars or amino acids is inactive. Conjugation to sugars is mediated through UDP‐glucosyl transferase (UGT) enzymes (Szerszen *et al*., 1994) and to amino acids using the Gretchen Hagen 3 (GH3) family of IAA‐amido synthetases (Hagen *et al*., 1991; Staswick *et al*., 2005). Importantly, conjugation with most amino acids is reversible enabling free IAA to be regenerated by amidohydrolases such as IAA‐LEUCINE RESISTANT 1‐like (IRL1) and IAA‐ALANINE RESISTANT 3 (IAR3) (Bartel & Fink, 1995; Davies *et al*., 1999). Two exceptions, IAA‐Asp and IAA‐Glu, provide a fourth level of regulation (Fig. 3a, green) because they trigger IAA oxidation and subsequent degradation (Ludwig‐Muller, 2011). Although not exclusively a consequence of conjugation with Asp and Glu, auxin is oxidised and degraded by the enzyme DIOXYGENASE FOR AUXIN OXIDATION (DAO) (Zhao *et al*., 2013). Changes to the auxin conjugation machinery have been achieved by overexpression of conjugating enzymes (Romano *et al*., 1991) or by multiple gene knockouts of conjugated IAA hydrolases (Spiess *et al*., 2014). Both manipulations led to pleiotropic defects associated with either a decrease or increase in active auxin, including defects in vascular differentiation (for overexpression in tobacco) or a fragmented vascular network (for multiple knockouts in Arabidopsis). Interestingly, in Arabidopsis, loss of function of IAA‐conjugate hydrolases led to an increase in the expression of genes encoding IAA biosynthetic enzymes (Spiess *et al*., 2014). Regulated interplay between auxin synthesis, conjugation and degradation therefore influences where, when and how much auxin accumulates in cells of the leaf, and in so doing must play a role in leaf venation patterning, but because auxin can move intercellularly this is only part of the picture.

### III..3 Transport

The bulk of auxin is synthesised in source organs and is then transported, either passively or actively, to elsewhere in the plant or to other locations within the same organ. Passive long‐range movement occurs via the phloem (Goldsmith *et al*., 1974), whereas passive short‐range movement occurs by diffusion across the plasma membrane or through plasmodesmata (Han *et al*., 2014; Gao *et al*., 2020). Because auxin is mildly acidic, passive transport across membranes is affected by local pH (Fig. 3b). In the acidic milieu of the apoplast (*c*. pH 5.5), auxin is partially protonated to form IAAH but in the cytosol (*c*. pH 7.0) unprotonated IAA^‐^ predominates. Neutrally charged IAAH can diffuse through the lipid bilayer from the apoplast to the cytosol whereas IAA^−^ cannot cross from the cytoplasm to the apoplast. Passive movement therefore contributes to long‐range source‐to‐sink translocation and to short‐range cellular influx, but cellular efflux occurs almost exclusively by active transport. Interestingly, diffusion through plasmodesmata may have a ‘nonpassive’ component, with directional flux achieved by unequal deposition of callose in plasmodesmata of different cellular axes (Gao *et al*., 2020). Although it is unclear how plasmodesmatal and transporter‐mediated auxin fluxes are coordinated, active auxin transport is required for a plethora of cellular and developmental processes including phyllotaxis, organ initiation and vascular patterning (reviewed in Petrasek & Friml, 2009; Zazimalova *et al*., 2010; Skalický *et al*., 2018).

Various auxin transporters have been isolated including AUXIN‐RESISTANT1/LIKE‐AUX (AUX1/LAX), PIN‐FORMED (PIN), PIN‐LIKES (PILS) and ATP‐binding‐cassette B (ABCB) family proteins (reviewed in Petrasek & Friml, 2009; Zazimalova *et al*., 2010; Balzan *et al*., 2014) (Fig. 3b). Plasma membrane (PM)‐localised AUX1/LAX transporters act to facilitate movement of auxin into the cytoplasm (Peret *et al*., 2012). By contrast, PIN proteins act as auxin efflux carriers at the PM and as both efflux and influx carriers at the endoplasmic reticulum (ER). All PIN proteins consist of five transmembrane helices at both the N‐ and C‐termini that are separated by a cytoplasmic hydrophilic loop (HL), with ‘canonical’ PM‐localised PINs that have long hydrophilic loops and noncanonical ER‐localised PINs that have short hydrophilic loops (Viaene *et al*., 2013; Zhang *et al*., 2020a,b). Intermediate length HLs have been associated with localisation at both the PM and the ER (Simon *et al*., 2016). As for noncanonical PINs, PILS are localised at the ER where they act to maintain intracellular auxin homeostasis, probably by sequestering auxin in the ER and therefore regulating the amount of auxin available for diffusion into the nucleus (Barbez *et al*., 2012). The role of ABCB transporters, which localise nonpolarly at the PM, is more complicated than that of AUX/LAX, PIN and PILS because only some family members have auxin transport ability (Noh *et al*., 2001; Geisler *et al*., 2005; Lewis *et al*., 2009). Of those, some are efflux carriers (Geisler *et al*., 2005), whereas others can either import or export depending on auxin concentration in the cell (Kamimoto *et al*., 2012; Kubes *et al*., 2012). Clearly, coordinated regulation of these different transporter types, both intercellularly and intracellularly, is necessary to modulate auxin levels and activity within plant tissues.

Whereas ER‐localised PINs and PILS maintain intracellular auxin homeostasis (Mravec *et al*., 2009; Ding *et al*., 2012), canonical PM‐localised PINs play a pivotal role in polar auxin transport (PAT) and therefore in developmental processes that involve PAT, such as venation patterning. In Arabidopsis roots, the polar localisation of PINs is regulated by a combination of endocytic recycling that delivers PIN proteins to the basal PM, the formation of protein clusters within the basal PM to prevent lateral diffusion, and localised clathrin‐mediated endocytosis at lateral PMs (Kleine‐Vehn *et al*., 2008, 2011). Intriguingly, an ABCB protein stabilises PIN1 in specific domains of the PM, leading to enhanced auxin flux and reduced PIN1 recycling (Titapiwatanakun *et al*., 2009). Once localised, PIN activity is regulated by phosphorylation of specific residues within the HL by either PINOID (PID) or D6 PROTEIN KINASE (D6PK), with D6PK being localised specifically at the basal PM (reviewed in Barbosa *et al*., 2018). Delivery of PIN and D6PK to the basal PM is mediated by ADP‐ribosylation factor GTP‐exchange factors (ARF‐GEFs) such as GNOM and, in both cases, the process is induced by auxin (Sauer *et al*., 2006; Kleine‐Vehn *et al*., 2008; Barbosa *et al*., 2014). In this way, PAT is reinforced by auxin itself, generating a feedback loop that plays a role in the specification and elaboration of leaf veins (see Section IV for further details). Recent work in Arabidopsis has shed light on the role of these regulators, specifically GNOM, in the context of cell polarity in leaves (Verna *et al*., 2019). Phenotypic characterisation of loss‐of‐function mutations in single or multiple *PIN* genes, with or without mutations in *GNOM*, suggested that GNOM controls both auxin transport and signalling during vein formation and patterning, a role that has not yet been uncovered in Arabidopsis roots (Verna *et al*., 2019). These findings suggest that the mechanism and control of cell polarity during vein formation and patterning is likely to differ between root and shoot, despite shared molecular players.

### III..4 Signalling

The canonical auxin signalling pathway induces the transcription of genes that contain auxin response elements (AuxREs) in their promoters (Fig. 3c) (reviewed in Lavy & Estelle, 2016; Weijers & Wagner, 2016; Leyser, 2018). AuxREs are bound by dimers of auxin response factor (ARF) transcription factors that contain a DNA‐binding domain and adjacent dimerisation domain at the N terminus, a central transactivation or repressor domain, and a C‐terminal type I/II Phox and Bem1 (PB1) domain. At low auxin concentrations, Aux/IAA transcriptional repressors interact with ARFs via the PB1 domain and inhibit ARF activity (Fig. 3c) (Korasick *et al*., 2014) whereas, at high concentrations, auxin binds both to a degron motif in domain II (DII) of the Aux/IAA protein and to a F‐box protein of the TIR1/AFB family. The F‐box is associated with an E2 ubiquitin ligase, RING box protein 1 (RBX1), cullin 1 (CUL1) and S‐phase kinase associated protein 1 (SKP1), which together comprise the E3 ubiquitin ligase SCF^TIR/AFB^ complex (Ulmasov *et al*., 1997, 1997,1997, 1997; Boer *et al*., 2014; Lavy & Estelle, 2016). The auxin‐mediated interaction between TIR1/AFB and Aux/IAA leads to polyubiquitination of the Aux/IAA by the SCF complex and subsequent degradation by the proteasome (Gray *et al*., 2001; Dos Santos Maraschin *et al*., 2009). ARFs are therefore de‐repressed allowing the transactivation of auxin response genes (Fig. 3c) (Dharmasiri *et al*., 2005; Kepinski & Leyser, 2005; Tan *et al*., 2007; Calderón Villalobos *et al*., 2012). Notably, several ARFs act as transcriptional repressors rather than activators, but their mode of action is unclear (Gray *et al*., 2001; Dos Santos Maraschin *et al*., 2009). Because each component of the auxin signalling pathway is encoded by a member of a multigene family (e.g. in Arabidopsis there are 6 AFB, 29 Aux/IAA and 23 ARF proteins), and different family members display different interaction affinities and/or stability, the ‘canonical’ auxin pathway has multiple forms both within and between species, each of which can be recruited into different developmental contexts (reviewed in Leyser, 2018).

### III..5 *In vivo* detection

**Fig. 4:**
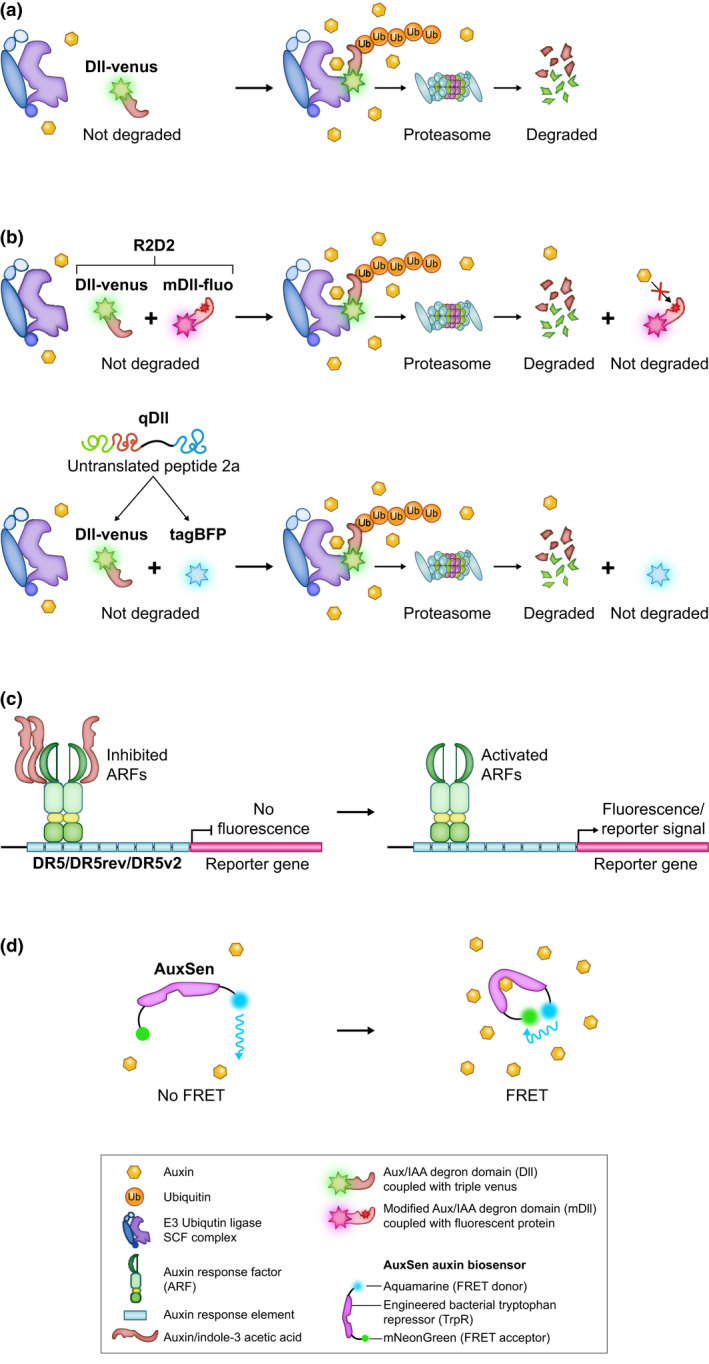
Nondestructive tools to study auxin. (a) Nonratiometric reporter constructs contain DII fused to a fluorescent reporter protein, generally triple‐Venus. Auxin signalling is detected when the DII domain (and therefore the fused reporter protein) is degraded. (b) Ratiometric reporter constructs additionally contain a nondegradable fluorescent reporter that is generated either by fusing a modified DII (mDII) domain that is not targeted for degradation by auxin with a second fluorescent protein (R2D2), or by linking tagBlue‐Fluorescent‐Protein (tagBFP) to DII‐Venus via a 2A peptide (qDII). (c) Transcriptional response reporters use artificial promoters (DR5, DR5rev and DR5v2) containing several auxin response element (AuxRE) repeats to drive the expression of reporters such as GUS or fluorescent proteins. (d) AuxSen is a synthetic auxin sensor that combines an engineered bacterial tryptophan repressor (TrpR) with a pair of Fӧrster resonance energy transfer (FRET) fluorophores. When auxin binds to the TrpR it causes a conformational change that brings the donor and acceptor fluorophores together resulting in FRET.

Given the complexity of auxin signalling and response pathways, elucidating the role of the hormone in any particular developmental process requires an understanding of how spatial and temporal patterns of activity differ within and between tissues. To this end, several nondestructive tools have been developed to study auxin signalling *in vivo*. For example, signalling activity can be detected using fluorescent reporters that are fused to the DII degron sequence of Aux/IAA proteins (Brunoud *et al*., 2012) (Fig. 4a). In low auxin, DII is intact and fluorescence can be detected but when auxin levels are high, auxin binding to DII triggers degradation of the fusion protein and the fluorescence signal decreases. Quantification of the signalling response is enabled by the use of ratiometric DII reporters in which either the same promoter is used to drive expression of both the degradable DII and the auxin insensitive nondegradable mDII reporters (Liao *et al*., 2015). Alternatively, the degradable DII‐mVenus reporter is linked to tagBlue‐Fluorescent‐Protein (tagBFP) by a 2A peptide that causes ribosome skipping and therefore translation of the two proteins as individual entities (Wend *et al*., 2013; Galvan‐Ampudia *et al*., 2020) (Fig. 4b). Due to their ability to bind auxin, DII reporters have been used to infer auxin levels *in planta* but binding of auxin to endogenous DII sequences requires the TIR1/AFB co‐receptor and degradation requires the SCF^TIR/AFB^ complex, both of which can vary within and between tissues. As such, DII reporter activity is not representative of auxin level *per se*. A second approach to monitor auxin distribution profiles uses transcriptional response reporters in which artificial promoters (DR5, DR5rev and DR5v2) that contain several AuxRE repeats fused to the minimal CaMV35s promoter are used to drive β‐glucuronidase (GUS) or fluorescent reporter gene expression (Ulmasov *et al*., 1997, 1997,1997, 1997; Liao *et al*., 2015). DR5 variants with different sensitivities are engineered by varying both the AuxRE motifs used and the configuration/spacing between, which in turn impacts on the ability of different ARF dimers to bind (Boer *et al*., 2014). In all cases, reporter gene activity is detected in regions of high auxin (Fig. 4c), but again, relative activity cannot be used as a proxy for auxin level *per se* because any response depends on activity of the upstream signalling machinery. Only AuxSEN, a direct auxin biosensor that uses an engineered bacterial tryptophan repressor (TrpR) coupled with fluorescence resonance energy transfer (FRET) allows the direct detection of auxin *in vivo* (Herud‐Sikimić *et al*., 2021) (Fig. 4d), but even with AuxSEN there may be limitations such as the range of concentrations that can be detected (reviewed in Balcerowicz *et al*., 2021). Combined use of all three reporter systems, however, would allow an evaluation of the spatial distribution of auxin (AuxSEN), of auxin signalling inputs (activation) (DII) and of auxin signalling outputs (response) (DR5), plus any temporal delay between auxin input and transcriptional response can be monitored. Because much of our understanding of how auxin influences leaf venation patterning is currently based on the use of just DII and DR5 reporters, with different combinations of reporter systems used in different experimental contexts, any comparison between studies requires careful interpretation.

### III..6 Evolution

Given the number and combinatorial complexity of components involved in auxin signalling and response pathways in angiosperms, it is reasonable to ask whether any particular component(s) evolved in the last common ancestor of lycophytes and euphyllophytes that could have facilitated deployment of the auxin pathway for the development of vascular structures. Genomic (Rensing *et al*., 2008; Bowman *et al*., 2017) and genetic analyses have shown that synthesis via the TAA/YUC pathway (Thelander *et al*., 2018), polar transport via PIN1 (Bennett *et al*., 2014; Zhang *et al*., 2020a,b) and signalling via AUX/IAA, ARF and TIR1/AFB proteins (Prigge *et al*., 2010; Flores‐Sandoval *et al*., 2015; Kato *et al*., 2015) occur in bryophytes, suggesting that the auxin pathway is likely to function in all land plants. However, genome data revealed potentially important gene duplication events as land plant groups diversified. For example, phylogenetic analyses indicated that different mechanisms of auxin transport appeared at different times, with sequences encoding the largely nonpolar ABCB, ER‐localised PILS and AUX1/LAX transporters found in chlorophyte algae, whereas those encoding polar PIN transporters were only found in the streptophyte lineage (charophyte algae plus land plants) (Barbez *et al*., 2012). In a second example, evolutionary trajectories of *AUX/IAA*, *ARF* and *TIR1/AFB* gene families revealed expansions of all three in the last common ancestor of euphyllophytes (Mutte *et al*., 2018). This observation suggests that the transition from single veins to complex venation patterns may have been facilitated by an enhanced capacity to spatially regulate auxin signalling in the developing leaf primordium. Finally, the number of *GH3* genes in the lycophyte *Selaginella moellendorffii* (i.e. 17) is similar to the 19 in Arabidopsis, but considerably higher than the two in the moss *Physcomitrella patens* (Banks *et al*., 2011), suggesting a possible expansion in the last common ancestor of vascular plants. Although the moss GH3 proteins conjugate IAA (Ludwig‐Muller *et al*., 2009), loss‐of‐function mutant phenotypes suggest that conjugation serves to protect plants from unfavourable growth conditions rather than to regulate developmental processes (Mittag *et al*., 2015). Gene duplication and neo‐functionalisation may therefore have allowed GH3‐mediated IAA conjugation to be incorporated into developmental programmes. Although a direct role for *GH3* genes in leaf venation patterning has not been demonstrated, in this scenario, the ability to spatially and temporally regulate the accumulation of bioactive auxin has evolved coincident with development of the land plant vascular system.

## IV. Ontogeny of leaf veins

Having considered the range of leaf venation patterns observed in land plants and the specifics of mechanisms that regulate auxin, we now move on to integrate our understanding of how the leaf venation network develops and of how auxin contributes to the process, identifying the unanswered questions that need to be addressed to reveal the genetic basis of the diversity observed.

### IV..1 Primordium initiation and development of the midvein

**Fig. 5:**
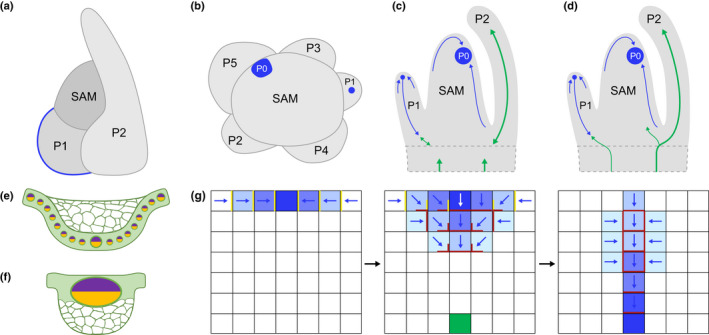
Schematic representation of primordium initiation and midvein formation in monocots and eudicots. (a) Sideways view of a monocot shoot apical meristem (SAM) with the two youngest (plastochron (P)1 and P2) leaf primordia encircling the apex and showing opposite (180°) phyllotaxy. The auxin provascular trace that marks the midvein position is depicted in blue in the P1 primordium. Any leaf can be described both by an invariant number that defines its position relative to the number of leaves that have expanded since germination (leaf 1 is always the oldest) and a variable number that defines developmental stage relative to initiation (P1 is always the youngest). (b) Top‐down view of a eudicot SAM with the five youngest (P1–P5) primordia showing spiral (137.5°) phyllotaxy. The auxin maximum in the SAM that marks P0 and the provascular trace that marks the midvein position in the P1 primordium are depicted in blue. (c, d) Longitudinal sections of shoot apices showing midvein formation in P1 and P2 primordia. Auxin traces, direction of flow and the maximum at P0 are shown in blue. In most monocots midvein (green) development is initiated *de novo* in the leaf primordium, extending both downwards into the shoot to join the stem vasculature and up into the leaf (c), whereas in eudicots the midvein extends from existing stem vasculature towards the leaf primordium (d). (e) Transverse section of a composite monocot midvein. The median vein, which is flanked by smaller veins on both sides, is positioned abaxially in photosynthetic tissue and clear parenchymatous cells are positioned adaxially. Sclerenchyma cells that form adaxially and abaxially are not shown. (f) Transverse section of a eudicot midvein. A single large vein is positioned adaxially in photosynthetic tissue and clear parenchymatous cells are positioned abaxially. Sclerenchyma cells that form adaxially and abaxially are not shown. (g) Direction of auxin flow during primordium initiation and midvein formation according to the dual polarisation model (adapted from data in Bayer *et al*. (2009)). Cells in the developing primordium are represented as a flat sheet of squares, with the top row representing the L1 epidermal layer. Relative concentrations of auxin are depicted by shades of blue (darker shade indicates a higher concentration) and the direction of auxin flow by blue arrows. Localisation of PIN1 transporters is indicated by yellow (*PIN1* in Arabidopsis but *SoPIN1* in angiosperms other than the Brassicaceae) or maroon (*PIN1*) lines. Existing stem vasculature is represented by the green square. At P0 (left panel) an auxin maximum is created in the L1 layer of the SAM as a consequence of lateral localisation of PIN1 and flux towards a convergence point. At early P1 (middle panel) auxin flow changes direction (white arrow), moving into cells below the convergence point and towards existing stem vasculature. At late P1 (right panel), canalisation towards the existing vasculature is reinforced by basal localisation of PIN1 and auxin flow within the provascular trace, and auxin levels in the trace are maintained by flow from adjacent cells in which PIN1 is localised laterally.

In both monocots and eudicots, leaves are formed from a multicellular, multilayered shoot apical meristem (SAM) (for general overview see Steeves & Sussex, 1989), with the switch from meristematic growth to determinate organ development initiated by the formation of an auxin maximum on the flanks of the SAM (Reinhardt *et al*., 2000, 2003; Benkova *et al*., 2003; Heisler *et al*., 2005; O'Connor *et al*., 2017). The spatial regulation of initiation events is geometrically precise, giving rise to recognisable phyllotactic patterns in which the angle between any two leaves is commonly 90°, 137.5° or 180°. However, temporal regulation is more variable both within and between species. Classically, the time interval between the initiation of successive leaf primordia is referred to as a plastochron (P), but the term has been adopted to denote the relative position of primordia (Sylvester *et al*., 1990). In this context, the location of the auxin maximum denotes an incipient primordium (P0), the smallest visible primordium is P1, the next largest is P2 and so on (Fig. 5a,b). Midvein development always starts before the emergence of P1, with PIN1 accumulation at the tip of the incipient primordium appearing progressively downwards towards the stem to mark the position of the provascular trace. Subsequently, the appearance of morphologically elongated cells and protophloem differentiation proceeds acropetally; these processes take place either through *de novo* specification in the leaf primordium (monocots, Fig. 5c), or through extension from the subtending stem vasculature (eudicots; Fig. 5d). In all cases examined, midvein position is predicted by auxin flux through a longitudinal file of cells referred to as the provascular trace (Fig. 5a–d). The establishment of auxin flux from the pool of cells on the flank of the SAM into the provascular trace is therefore the first step in the development of the leaf venation network.

A clear distinction between midvein development in monocots and eudicots has been revealed by the identification of midribless mutants in monocot species (Rao *et al*., 1988; Fladung, 1994; Yamaguchi *et al*., 2004; Strable *et al*., 2017; Richardson *et al*., 2020), but not in eudicot species. In wild‐type monocot leaves, the midvein is a composite structure comprised of some small veins either side of the median vein and achlorophyllous parenchyma cells (Fig. 5e). In all of the mutants, the midribless phenotype is caused by a failure to develop the composite midvein at P2, as opposed to a failure to specify the median vein. Causative mutations are found in the *DROOPING LEAF* genes of maize and rice that encode members of the YABBY family (Yamaguchi *et al*., 2004; Strable *et al*., 2017) and in an AUX/IAA family member (*ZmIAA28*) in maize (Richardson *et al*., 2020). *YABBY* genes promote organ growth and expansion (Eshed *et al*., 2004) in both monocots and eudicots, particularly during carpel development (Bowman & Smyth, 1999; Yamaguchi *et al*., 2004; Strable *et al*., 2017), but the recruitment of *DROOPING LEAF* for localised development and proliferation along the midline of the leaf primordium is monocot‐specific. Crucially, in the absence of this proliferation the median vein appears anatomically similar to adjacent lateral veins, all of which develop normally. This observation suggests that, in monocots, the development of higher order (lateral) veins is not dependent on the formation of the midvein, and that the median vein is inherently equivalent to a lateral vein.

Although midveins in monocots and eudicots are fundamentally different both in terms of ontogeny (initiate *de novo* vs extend from the stem vasculature; Fig. 5c,d) and anatomy (composite vs single; Fig. 5e,f), formation of both is preceded by an auxin‐associated provascular trace. Models to explain how this trace forms have been developed over many years, with the canalisation hypothesis being the first (Sachs, 1969, 1981). Canalisation assumes that auxin moves towards a sink where levels are low and that flux through a cell is reinforced in the direction of travel. Together these parameters create a unidirectional flow, as seen in the provascular trace. Initially, canalisation models predicted low auxin levels in trace cells (Mitchison, 1980; Rolland‐Lagan & Prusinkiewicz, 2005), whereas in Arabidopsis higher auxin response levels (as detected by DR5 activity) are detected in the trace than in surrounding cells (Scarpella *et al*., 2006). This discrepancy was resolved when the assumption that different areas of the PM compete for free PIN1 carriers was incorporated into models (Feugier *et al*., 2005). Alternative models proposed that the auxin maximum at P0 is formed because PIN1 transporters in epidermal cells of the SAM are positioned laterally and are oriented towards cells with higher auxin concentration (Jonsson *et al*., 2006; Smith *et al*., 2006). This leads to a convergence point of high auxin that marks the site of leaf initiation. Shortly after leaf initiation, PIN1 proteins are reoriented to the basal side in some of the high auxin containing epidermal cells, channelling auxin into the internal cell layers (Scarpella *et al*., 2006; Bayer *et al*., 2009). Flux through these cells reinforces the basal polarity of PIN1 localisation, narrowing the provascular trace to align with vasculature in the subtending shoot.

Until recently, models simulating either up the gradient (UTG) (Jonsson *et al*., 2006; Smith *et al*., 2006; Merks *et al*., 2007) or with the flux (WTF) (Stoma *et al*., 2008) auxin movement failed to explain both leaf primordium and midvein positioning. For example, UTG flux could only account for the provascular trace if subepidermal cells below the auxin maximum at least transiently exhibited higher auxin levels than the overlying epidermis (Merks *et al*., 2007), which has not been observed experimentally (Scarpella *et al*., 2006). The first model to simulate both leaf primordium and midvein positioning whilst accounting for all experimental data, proposed a combination of UTG and WTF mechanisms (Bayer *et al*., 2009) (Fig. 5g). In this dual polarisation model, UTG flux explained primordium positioning, whereas a combination of both UTG and WTF explained midvein positioning, with WTF movement channelling auxin into internal cells of the primordium and then the provascular trace being narrowed and maintained by UTG flux from neighbouring cells in which PIN1 is localised laterally. More recent models explained both processes with a single flux‐based mechanism that is based on antagonistic (canalisation) or synergistic (convergence) relationships between auxin influx and efflux carriers within cells – but unknown factors had to be invoked to explain how veins were guided towards existing vasculature (Cieslak *et al*., 2015; Hartmann *et al*., 2019). Given that epidermal auxin convergence points were proposed as the trigger for auxin movement into internal cells (Scarpella *et al*., 2006), but recent experiments revealed that they are neither necessary or sufficient for midvein formation in Arabidopsis (Govindaraju *et al*., 2020; Lavania *et al*., 2021), unknown components of auxin flux mechanisms obviously exist. In this context, both experimental and modelling approaches have suggested that mechanical stresses mediated by microtubule and cellulose microfibril dynamics play a role in both UTG and WTF movement of auxin, and it is becoming increasingly clear that the significance of auxin‐driven reorientation of PIN1 proteins during leaf primordium and midvein formation needs to be reevaluated in a more complex framework than previously considered (recently discussed and reviewed in Heisler, 2021; ten Tusscher, 2021; Vernoux *et al*., 2021).

Given that PIN1 polar localisation is associated with both phyllotactic patterns and midvein positioning, it is important to understand whether different PIN1 family members have specific roles. In monocots, auxin flow across the SAM is mediated by Sister‐of‐PIN (SoPIN) proteins (O'Connor *et al*., 2014, 2017), whereas in Arabidopsis PIN1 fulfils this role (Reinhardt *et al*., 2000, 2003; Benkova *et al*., 2003). This distinction is not a monocot–eudicot difference because SoPIN1 homologues also regulate leaf positioning in tomato (Martinez *et al*., 2016). Instead, it reflects the evolutionary loss of *SoPIN1* genes in the Brassicaceae (including Arabidopsis) (O'Connor *et al*., 2014). Whether it is SoPIN1 or PIN1 that specifies phyllotactic patterns impacts on how PIN1 is regulated to specify the midvein. In maize and *Brachypodium distachyon*, *SoPIN1* expression is associated with leaf positioning, whereas *PIN1a* expression marks the provascular trace (Carraro *et al*., 2006; Gallavotti *et al*., 2008; Lee *et al*., 2009; O'Connor *et al*., 2014; Johnston *et al*., 2015). *PIN1a* expression is therefore regulated solely in the context of venation patterning, whereas the *PIN1* expression domain in Arabidopsis is dynamically altered between P0 and P1 to facilitate both leaf and midvein positioning (Scarpella *et al*., 2006). Interestingly, PINs are not the only example in which Arabidopsis represents an outlier. For example, mutations in a single *WUSCHEL (WUS)‐RELATED HOMEOBOX* (*WOX*) gene perturb leaf development and vein patterning in the eudicots *Medicago truncatula* and *Nicotiana sylvestris* (Tadege *et al*., 2011), whereas mutations in multiple WOX genes are required to perturb leaf development in Arabidopsis (Z. Zhang *et al*., 2020; Zhang *et al*., 2020a,b). As such, although many developmental genes required for leaf venation patterning are best characterised in Arabidopsis, the regulatory mechanisms identified may not be representative of those in other angiosperms.

### IV..2 Primordium growth and development of the venation network

Once the midvein position in the leaf primordium has been established, and elongation and cell division of the associated procambial cells has begun, the specification of procambial cells that will form subsequent vein ranks proceeds via two distinct routes in monocots and eudicots. In monocots, elongated procambial cells that initiate secondary vein formation appear with no connection to the midvein or to the stem vasculature, whereas in eudicots there is a physical connection to the midvein, which itself is an extension of the stem vasculature. This feature is extrapolated through all vein ranks such that veins in monocots initially appear in physical isolation (although they can anastomose later in development), whereas veins in eudicots appear progressively with tertiary veins extending from secondary veins and so on. Given these different trajectories, relationships between growth, axis formation and venation patterning in monocots and eudicots are discussed separately below.

#### Patterning in monocot leaves

**Fig. 6:**
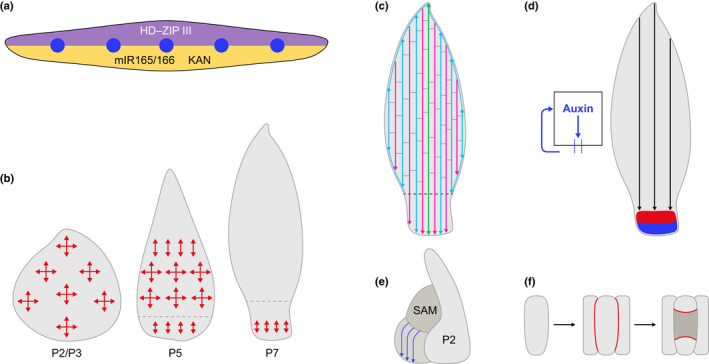
Schematic representation of cell division dynamics and vein patterning in monocot leaves. (a) The adaxial–abaxial axis is specified at plastochron 1 (P1) by expression of HD‐ZIP III genes adaxially (purple) and miR165/166 and KANADI genes abaxially (orange). Auxin‐mediated provascular traces (blue) traverse the region where the two domains juxtapose, prepatterning the adaxial–abaxial axis in the trace. (b) Cell divisions occur both transversely (vertical red arrows) and longitudinally (horizontal red arrows) throughout P2/P3 primordia. At P5, the ligule (dashed grey line) is visible and division has ceased at the tip of the blade. All cells within the sheath and some within the blade divide exclusively in the transverse orientation to increase leaf length. At P7, divisions are restricted to the base of the sheath. Note that division patterns have not been observed directly in internal leaf tissues but have been deduced from cell lineage analyses (Poethig, 1984) and from direct observation of divisions in the leaf epidermis (Sylvester *et al*., 1990). (c) The proximo‐distal and medio‐lateral patterns of lateral (turquoise), intermediate (pink) and transverse (grey) veins in the mature leaf results in part as a consequence of the cell division dynamics illustrated in (b). That is, once divisions cease at the tip of the primordium and divisions in the sheath are exclusively transverse, some of the lateral veins that initiate at the base of the blade can neither fully extend to the tip of the blade nor extend downwards into the sheath. Similarly, some of the intermediate veins that extend basipetally cannot initiate at the leaf tip nor extend into the sheath and instead have to start and end closer to the middle of the leaf. This leads to anastomoses at the base and tip of the blade and therefore to venation patterns that are distinct from those seen in the middle of the blade. (d) The development of a parallel venation pattern can be simulated with three parameters: basal auxin efflux and reinforcement of auxin levels in cells as a consequence of that flux, an auxin sink and a restricted cell division zone at the leaf base. Only basipetal vein development can be simulated in this way. (e) Auxin provascular traces (blue) simultaneously mark the future position of lateral veins at P1. (f) Single procambial initials normally undergo two rounds of asymmetric divisions to yield a group of five cells. The initial first divides twice longitudinally (once to the left and once to the right) to yield daughter cells that will develop into the sheath cells on the lateral sides of the vein. The initial then divides twice periclinally to produce daughters that will form the sheath cells above and below the vein. The central cell (dark grey) goes on to divide and differentiate into xylem and phloem tissues of the vein, defining the procambial lineage. The upper, lower and lateral cells divide to increase the circumference of the sheath as the vein increases in size. Red lines depict new cell walls following each division round. SAM, shoot apical meristem.

Monocot leaf development is initiated when KNOTTED1‐like homeobox (KNOX) gene expression is suppressed at P0 (Jackson *et al*., 1994; Smith *et al*., 1995) and in maize this process is inhibited when PAT is inhibited in cultured shoot apices (Scanlon, 2003; reviewed in Conklin *et al*., 2019). Formation of an auxin maximum at P0 is therefore necessary both for positioning new primordia and for inducing the switch from meristematic (KNOX on) to leaf (KNOX off) cell fate. As the leaf primordium forms it has inherent adaxial–abaxial polarity in that it emerges as a flattened structure with the adaxial side abutting the SAM. Genetic analyses of rolled leaf mutants in maize and rice revealed that the mechanism of adaxial–abaxial axis specification is conserved with that first identified in Arabidopsis (Juarez *et al*., 2004a,b; Candela *et al*., 2008; Zhang *et al*., 2009, 2021). Briefly, adaxial identity is conferred by HD‐ZIP III transcription factor family members and abaxial identity by both KANADI transcription factors and microRNA 165/166‐mediated turnover of HD‐ZIP III transcripts (Fig. 6a). The HD‐ZIP III genes *Rolled1* (*Rld1*) (Juarez *et al*., 2004a) and *LATERAL FLORET 1* (Zhang *et al*., 2021) encode the adaxialising factors in maize and rice respectively, with the KANADI genes *Milkweedpod1* (Candela *et al*., 2008) and *SHALLOT‐LIKE 1* encoding the corresponding abaxialising factors. Loss‐ and gain‐of‐function mutations in any of these genes cause leaf rolling and ectopic positioning of xylem (if adaxialised) or phloem (in abaxialised) within leaf veins. The coincidence of rolled leaf and aberrant xylem–phloem phenotypes in mutant plants suggests that adaxial–abaxial polarity within monocot leaf veins may be prepatterned by the juxtaposition of HD‐ZIPIII and KAN/miR165/166 activity within the initiating leaf primordium (reviewed in Satterlee & Scanlon, 2019). This suggestion is supported by the observation that *Rld1* expression is restricted to the adaxial leaf domain in maize at P1 (Juarez *et al*., 2004a), whereas the HD‐ZIP III genes in Arabidopsis are initially expressed throughout the leaf primordium, only becoming adaxialised between P2 and P3 (McConnell *et al*., 2001). Such a prepatterning mechanism might be necessary to specify adaxial–abaxial polarity in monocot leaf veins, because they initiate *de novo* in the primordium, whereas eudicot leaf veins extend from the existing stem and/or leaf vasculature and therefore the adaxial–abaxial axis is already formed.

Positioning and development of veins in the proximo‐distal and medio‐lateral leaf axes is interconnected with growth and cell divisions in the primordium, the timing of which is best characterised in maize (Sharman, 1942; Poethig, 1984; Sylvester *et al*., 1990; Smith *et al*., 1996). Cell divisions occur throughout the primordium between P2 and P3, with transverse (contributing to leaf length) and longitudinal (contributing to leaf width) divisions approximately equal in number and the primordium ovate in shape (Fig. 6b). During P3, the preligular band develops and divisions cease at the primordium tip. Divisions become progressively more restricted to the base through to P7 and by P8 growth is mediated solely by cell expansion. With respect to vein formation, the files of elongated procambial cells associated with lateral veins develop between P2 and P4, extending downwards from the base of the primordium to connect with the stem vasculature and acropetally (from base to tip) within the primordium (Carraro *et al*., 2006; Lee *et al*., 2009; Johnston *et al*., 2015). Files associated with intermediate veins extend basipetally (from tip to base) from P3 to P5, with rank 1, but not rank 2, intermediates extending beyond the ligule into the sheath (Russell & Evert, 1985). Patterning of the intermediate veins is dependent on PAT, as demonstrated by sheath‐like patterns in maize leaf blades after exposure to PAT inhibitors at P4/P5 (Tsiantis *et al*., 1999) and altered patterns in rice mutants with reduced auxin transport capacity (Scarpella *et al*., 2002; Qi *et al*., 2008). By the end of P5, basipetal formation of transverse veins and anastomoses of veins at the tip and base of the blade close the striated network. This sequence of events is similar in other monocots such as wheat (Sharman & Hitch, 1967; Blackman, 1971), barley (Dannenhoffer & Evert, 1994) and sugarcane (Colbert & Evert, 1982). Mapping spatial and temporal patterns of cell division onto leaf vein ontogeny in maize reveals that both the acropetal extension of lateral veins (therefore length in the proximo‐distal axis) and the number of intermediate veins initiated (therefore vein spacing in the medio‐lateral axis) must be constrained from late P3 onwards by basipetal movement of the cell cycle arrest front (Fig. 6c). The topology of the leaf venation network could therefore be determined indirectly via coordinated regulation of cell divisions across the developing primordium, a scenario that needs experimental validation.

Cell division dynamics can partially explain venation patterns in monocot leaves but how ground meristem cells in the primordium become specified to form procambium remains enigmatic. Mathematical models have simulated parallel venation patterns using three parameters: basipetal auxin flux reinforcing auxin levels in a cell, an auxin sink across the entire leaf base, and a restricted cell division zone at the base (Fujita & Mochizuki, 2006) (Fig. 6d). However, this model only simulates the formation of basipetally developing veins and therefore cannot explain the acropetal development of lateral veins. Notably, the future position of at least some lateral veins in maize is predicted by PIN1‐marked provascular traces that form alongside the midvein trace during P1, extending basipetally from auxin convergence points at the tip of the primordium (Carraro *et al*., 2006; Lee *et al*., 2009; Johnston *et al*., 2015) (Fig. 6e). This pattern differs from the one described in the eudicot model Arabidopsis, in which provascular traces for secondary veins are not observed until after the midvein has formed, with the midvein itself proposed to act as the sink to guide auxin canalisation from convergence points at the leaf margin into secondary provascular traces (Scarpella *et al*., 2006). Together these observations reinforce the suggestion that the median vein in monocots is equivalent to a lateral vein, and also invoke the presence of a prepatterning mechanism in the medio‐lateral leaf axis that imparts competence to respond to auxin in some ground meristem cells of the P1 primordium and not others, and therefore determines vein density.

Cells that become specified to form procambium are recognisable from neighbouring ground meristem cells by an elongated shape. We refer to these single cells, that arise *de novo* in monocot leaf primordia, as procambial initials (Sedelnikova *et al*., 2018). Molecular markers of these cells and/or their derivatives are lacking, the one exception being an HD‐Zip II protein (OsHOX1) that promotes auxin efflux in rice (Scarpella *et al*., 2002). *Oshox1* is expressed shortly after cells become recognisable as procambium and expression persists throughout vascular development (Scarpella *et al*., 2000, 2005). The lack of markers in monocots arises partly because mutants with altered venation patterns generally have pleiotropic phenotypes, with venation defects secondary to other perturbations (Scarpella *et al*., 2003; Qi *et al*., 2008; Rizal *et al*., 2015). For example, mutations that alter leaf width lead to an increase (narrow leaves) or decrease (wide leaves) in vein density across the medio‐lateral leaf axis (Smillie *et al*., 2012). Despite the lack of molecular markers, cell lineage and histological analyses have elucidated early ontogenetic events in maize, rice and other grasses. Briefly, veins and the sheath cells that surround them develop from procambial initials that are specified in the innermost layer of the leaf primordium (Langdale *et al*., 1989). Most veins develop acropetally or basipetally from a single file of initials, although veins can be produced from the coordinated activity of two adjacent initials (Langdale *et al*., 1989; Bosabalidis *et al*., 1994; Sud & Dengler, 2000). In maize, single initials undergo two rounds of asymmetric divisions to produce a central cell that defines the vein‐forming procambial lineage and four cells that develop into the surrounding sheath cells (Fig. 6f) (Sharman, 1942; Bosabalidis *et al*., 1994). Although some species‐specific differences have been observed, for example bundle sheath cells in maize are derived from procambial initials, whereas those in rice are derived from the ground meristem (Sakaguchi & Fukuda, 2008), the general pattern of divisions is conserved and the sheath cell layer closest to the vein (bundle sheath in maize but mestome in rice) develops from procambial initials. At present, however, it is still unknown how procambial initials are specified at regular intervals across the medio‐lateral leaf axis and whether the process is the same for cells that are specified for lateral vein formation at the base of the P1/P2 primordium and cells that are specified for intermediate vein formation at the tip of the P3/P4 primordium.

In the next section we assess how the patterning processes operating in monocots, that is *de novo* specification of procambium in the ground meristem of the leaf primordium, prepatterning of the adaxial–abaxial axis in vascular strands (and therefore the arrangement of xylem and phloem), midvein‐independent induction of auxin traces for lateral veins (indicative of a further prepattern in the medio‐lateral axis), and constraints on the area of the venation network imposed by cell division arrest, compared with those identified in eudicots.

#### Patterning in eudicot leaves

As in monocots, the emergence of the eudicot leaf primordium is facilitated by polarised expression of *PIN1* and the repression of KNOX gene expression in a small cohort of founder cells (Long *et al*., 1996; reviewed in Conklin *et al*., 2019). PAT inhibitors prevent leaf initiation and local application of auxin at the site of primordium emergence can overcome this inhibition (Reinhardt *et al*., 2000). Also as in monocots, PAT is similarly involved in midvein positioning and in secondary and higher vein formation in the leaf, with plants grown on medium supplemented with PAT inhibitors displaying an increased number of secondary veins with misaligned vascular cells files, a phenotype also observed in *pin1* mutants (Mattsson *et al*., 1999; Sieburth, 1999). The increase in secondary vein number is accompanied by a reduction in the number of higher rank veins (Huang *et al*., 2017) revealing a central role for regulated auxin efflux in leaf vein patterning (see also Ravichandran *et al*., 2020; Lavania *et al*., 2021).

**Fig. 7:**
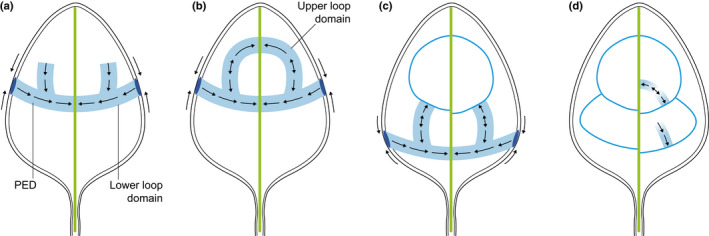
Prepatterning of secondary and higher rank veins in *Arabidopsis thaliana* driven by *PIN1* expression domains (PED). (a) After the provascular trace of the midvein is formed (green), PIN1 convergence points form on the epidermis at the leaf margin (dark blue), simultaneously with PEDs corresponding to the lower loop of secondary veins. (b) Following the lower loop domain connection to the midvein, an upper loop domain forms from the lower loop domain towards the distal part of the midvein. (c) Successive loops of second‐order veins form with a similar mechanism to that observed in (a, b), with the upper domain connecting to another loop or PED instead of the midvein. (d) Higher rank veins form following new PED extensions from existing PEDs or veins. The direction of the arrows indicates the polarity of PIN1 and can be used as a proxy to deduce the direction of auxin flow. Adapted from Scarpella *et al*. (2006) and Marcos & Berleth (2014).

*PIN1* expression domains (PED) define the position of provascular traces for both the midvein (Fig. 6d) and higher rank veins (Fig. 7) in Arabidopsis (Scarpella *et al*., 2006). Although most studies have characterised PEDs in cotyledons (which are specified during embryogenesis) and/or in the first pair of juvenile leaves, similar PED profiles are seen in adult leaves during the specification of primary veins (Scarpella *et al*., 2006). However, differences are observed during the specification of second‐order veins, consistent with the more reticulated venation network seen in adult leaves (Scarpella *et al*., 2006). Typically, once the provascular trace marking the midvein position is formed (Figs 6d, 7a), epidermal convergence points of *PIN1* expression appear at the leaf margin concomitantly to a PED (with PIN1 facing the existing vein) in the underlying ground tissues; PEDs progress towards the midvein marking the proximal part of a future vascular loop (Fig. 7a). The PED then gradually extends in cell files from this lower loop domain towards the distal midvein, marking the upper domain of the loop (Fig. 7b). Formation of other secondary vein loops follows a similar sequence of events but the upper domain connects by a bipolar cell to an existing distal loop instead of the midvein (Fig. 7c) (Scarpella *et al*., 2006). Interestingly, recent work has shown that epidermal accumulation of *PIN1* is dispensable; Arabidopsis seedlings that lack epidermal PIN1 develop venation patterns indistinguishable from the wild‐type (Govindaraju *et al*., 2020). However, PIN1 presence in the inner tissues is essential for normal leaf vein development (Govindaraju *et al*., 2020). Third and higher rank veins display a different PIN1 profile in that no epidermal *PIN1* expression is observed; instead expression appears in a single row of cells starting from an existing PED or mature vein with PIN1 oriented towards them (Fig. 7d). In this way, a connected PED is formed that consists of two files of cells with opposite PIN1 polarity that are connected by a bipolar cell, as in second‐order loops (Scarpella *et al*., 2006; Marcos & Berleth, 2014). PIN1‐mediated auxin flux therefore defines the positioning of provascular traces in both monocot and eudicot leaves, but monocot PEDs that mark different ranked veins can emerge simultaneously (e.g. for the midvein and adjacent laterals), suggesting the presence of a prepatterning mechanism, whereas those in Arabidopsis emerge progressively with existing PEDs/provascular traces guiding the position of new ones.

Whereas in monocots our understanding of the molecular mechanisms that regulate leaf venation patterning is limited to a role for PIN1 in the specification of provascular traces, studies in Arabidopsis have elucidated how those early events lead on to the development of procambium. First, auxin signalling at PIN1 convergence points leads to activation of the MONOPTEROS/ARF5 (MP) transcription factor, which in turn elevates *PIN1* expression (Przemeck *et al*., 1996; Hardtke & Berleth, 1998; Wenzel *et al*., 2007). In a second positive feedback loop, PIN1‐mediated canalisation of auxin through a narrow strand of preprocambial cells further reinforces *PIN1* expression (Sauer *et al*., 2006; Scarpella *et al*., 2006; Wenzel *et al*., 2007). Simultaneously, MP induces the expression of *ARABIDOPSIS THALIANA HOMEOBOX 8* (*ATHB8*), which encodes an HD‐ZIP III transcription factor that marks preprocambial cell identity and stabilises PIN1 localisation (Baima *et al*., 1995; Mattsson *et al*., 2003; Donner *et al*., 2009). These markers and others of preprocambial and procambial cells have allowed vascular development in the Arabidopsis leaf to be described more comprehensively than in any monocot leaf (Scarpella *et al*., 2004; Sawchuk *et al*., 2007). Specifically, preprocambial identity is first observed in cells near existing veins in the middle of the lamina and then extends toward the leaf margin. Procambium differentiation, recognisable by an elongated cell shape and expression of the molecular marker Q0990, then takes place simultaneously along an entire vein (Mattsson *et al*., 1999; Kang & Dengler, 2004; Scarpella *et al*., 2004; Sawchuk *et al*., 2007; Marcos & Berleth, 2014). Vein formation in Arabidopsis therefore occurs in three distinct steps: directional auxin flow through a file of provascular cells from margin to midvein, directional specification of preprocambium in that same file but from midvein to margin, and then simultaneous differentiation of procambium along the whole file.

Although the PIN1/MP/ATHB8 feedback loop is an important component of the venation patterning mechanism in Arabidopsis, other factors undoubtedly play a role. For example, *pin1,3;4;7* quadruple mutants display more perturbed venation patterns than *pin1* single mutants, indicating that these other PINs may work alongside PIN1 (Verna *et al*., 2019), and *AtHB8* gene expression does not mark preprocambial cells of higher rank veins that form later in leaf development (Kang & Dengler, 2004). PIN1 also acts redundantly with PIN6 to inhibit vein formation and vein connections, with PIN1 driving intercellular and PIN6 driving intracellular auxin transport. PIN8 further acts redundantly with PIN6 in both a PIN1‐dependent and ‐independent inhibition pathway, whereas PIN5 acts oppositely to promote vein formation in a PIN1‐independent manner (Sawchuk *et al*., 2013; Verna *et al*., 2015). Other known regulators of leaf venation patterning primarily influence auxin transport/signalling or *MP/ATHB8* expression (Koizumi *et al*., 2000; Petricka *et al*., 2008). For example, in accordance with a known role in auxin perception, *tir1,afb1,afb3* triple mutants display a strong venation defect in primary leaves with an apparent decrease in secondary vein number (Mazur *et al*., 2020). Mutations in genes encoding proteins required for PIN1 localisation, such as the vesicle trafficking protein GUANINE‐NUCLEOTIDE EXCHANGE FACTOR FOR ADP‐RIBOSYLATION‐FACTOR GTPASES (GNOM), the PH domain protein FORKED1 and the inositol polyphosphate 5′‐phosphatase COTYLEDON VASCULAR PATTERN, also cause vein patterning defects in leaves (Steynen & Schultz, 2003; Hou *et al*., 2010; Verna *et al*., 2019). Similarly, loss‐of‐function mutations in *SCARFACE/VASCULAR NETWORK DEFECTIVE‐3* (*SFC/VAN3*) disrupt PIN1 cycling as well as auxin signalling (Koizumi *et al*., 2005; Sieburth *et al*., 2006), leading to the formation of fragmented veins and discontinuous *ATHB8* expression (Deyholos *et al*., 2000; Koizumi *et al*., 2000; Scarpella *et al*., 2006). Auxin‐independent components of the regulatory pathway await discovery, but an extensive collection of mutants affecting vasculature development in eudicots already exists (reviewed in Scarpella & Meijer, 2004) and targeted genetic enhancer/suppressor screens and/or biochemical interactor screens could provide further candidates.

Most mutants with leaf venation defects also display changes to leaf shape, reflecting the relationship between vein patterning and the regulation of cell proliferation throughout the leaf (Petricka *et al*., 2008). For example, mutations in *WOX* gene family members in various eudicot species lead to leaf shape perturbations, notably a marked reduction in the leaf lamina, along with disrupted venation patterns (Vandenbussche *et al*., 2009; Tadege *et al*., 2011). In Arabidopsis, *WOX* genes have been shown to mediate growth of the leaf lamina through localised induction of *YUCCA* auxin biosynthetic genes, with auxin promoting cell proliferation and inhibiting differentiation (Z. Zhang *et al*., 2020; Zhang *et al*., 2020a,b). In this way, *WOX* gene expression profiles in the primordium influence leaf shape by regulating where and when cell proliferation occurs, and influence venation patterning by regulating where and when provascular traces will form. Consistently, continuous addition of new higher order veins and freely ending veinlets are observed as long as the leaf is growing (Kang & Dengler, 2004). During leaf growth, cell division becomes progressively restricted to the base and ceases completely at the tip, where endoreduplication causes cell enlargement (Donnelly *et al*., 1999). Loss‐of‐function mutations in genes encoding the GRAS transcription factors SHORTROOT (SHR) or SCARECROW (SCR) lead to a decrease in leaf cell number and to growth defects. Moreover, cell divisions cease prematurely suggesting that SHR and SCR act positively on the expression of cell cycle regulators (Dhondt *et al*., 2010). Silencing of such cell cycle regulators is also associated with altered leaf venation patterns (Kang *et al*., 2007; Marrocco *et al*., 2009), with increased cell proliferation leading to an increase in higher order veins and an early cessation of cell division causing a reduction in vein number (Kang *et al*., 2007). The induction of ectopic cell divisions in procambium and in ground tissues also perturbs venation patterning, with additional impacts on auxin responses (Wenzel *et al*., 2012). Notably, consistent with its proposed role in cambial cell proliferation and differentiation (Baima *et al*., 2001), *ATHB8* expression precedes the expression of core cell cycle genes. This observation highlights a probable positive feedback loop between the auxin regulatory network, cell divisions and vascular differentiation (Kang & Dengler, 2002; Marcos & Berleth, 2014).

Computational analyses have attempted to reproduce the formation of eudicot leaf venation patterns, with the goal of understanding the intricate relationships between cell proliferation, auxin signalling and the self‐organisation of venation patterns. Two conceptual frameworks have traditionally been used to study the relationship between auxin and venation patterning: reaction‐diffusion and canalisation, with computational modelling and experimental evidence alternatively favouring one or the other. Reaction‐diffusion, based on Turing's original reaction‐diffusion theory, was initially applied by Meinhardt to the broader concept of biological patterning (Meinhardt, 1976). The combined action of a slowly diffusing activator and a fast‐diffusing inhibitor can generate patterns that resemble a reticulated network, albeit quite far from those observed in Arabidopsis leaves. However, the reaction‐diffusion theory cannot, in its current form, explain how vascular strands can form *de novo* after wounding or exogenous auxin application. By contrast, substantial evidence is available in support of the canalisation hypothesis that was first proposed by Tsvi Sachs (Sachs, 1969, 1981) and then computationally elaborated by Mitchison (Mitchison, 1980, 1981). Canalisation proposes that auxin transport in a cell is positively regulated by the intensity of the auxin flux perceived, with cells polarising in the direction of the flux and therefore draining auxin from a source to a sink in discrete traces that will correspond to sites of vascular development. In its original form, the canalisation hypothesis accounted for the formation of linear veins and branched patterns. However, it could not explain the relationship between the basipetal movement of auxin and the acropetal differentiation of the midvein, nor the formation of closed loops of secondary veins. The former was addressed by assuming that acropetal development of the midvein could be sink rather than source‐driven and the latter by introducing discrete sources of auxin or a flux bifurcator (Feugier *et al*., 2005; Rolland‐Lagan & Prusinkiewicz, 2005). The flux bifurcator allows the extremity of one vascular trace to be attached to another, ultimately leading to a continuous auxin flux but it is notable that, particularly for Feugier’s flux bifurcator model, robust experimental evidence is lacking. Collectively, there is currently more support for the canalisation theory than for a reaction‐diffusion based mechanism but, in addition to auxin transport, other venation patterning models invoke roles for differential organ growth (Runions *et al*., 2005) or for localised auxin biosynthesis and the action of mechanical forces (Kneuper *et al*., 2020). As such, although the core assumptions of the canalisation theory still stand, the concept of canalisation driven solely by PAT is insufficient to explain the formation of complex venation networks. To date, the only models that can simulate both parallel venation in monocots and reticulate networks in eudicots incorporate the different growth dynamics observed in monocot and eudicot leaf primordia (in terms of position and duration of cell divisions), in addition to discrete sources of auxin production and auxin movement towards a sink by canalisation (Rolland‐Lagan & Prusinkiewicz, 2005; Feugier & Iwasa, 2006).

## V. Future perspectives

Understanding the mechanisms underlying leaf vein formation is of key importance given the impact of the vascular system on photosynthetic performance and leaf hydraulic capacity. Because of the potential to modify physiological performance, vein patterning mechanisms have gained much interest in the last few years as efforts are made to generate more climate‐resilient crops that have optimal photosynthetic rates and efficient distribution networks for both metabolites and water. Biophysical models have even attempted to determine which aspects of the leaf venation network optimise transport function and structural rigidity (Katifori *et al*., 2010; Ronellenfitsch, 2021). Here we provided an overview of the mechanisms of vascular patterning in extant land plants, with a particular focus on angiosperms and on key differences between monocots and eudicots with regard to vein ontogeny and patterning, and on the role of auxin in these processes. Experimental and computational work highlights the need for a better understanding of how specification, differentiation, proliferation and expansion processes are coordinated both within and between cells of the developing leaf vasculature. Additionally, there is a need to understand how the spatio‐temporal dynamics of auxin responses regulate the positioning of procambial initials, particularly in monocots in which procambium is specified *de novo* in the leaf and there is evidence of a prepatterning mechanism. To this end, advanced noninvasive techniques for live imaging of monocot leaf primordia need to be developed and the experimental data used to train computational models and simulations. Overall, there is enough evidence to suggest that knowledge gained in eudicots will only partially explain processes in monocots and therefore focussed investigations are needed to understand how developmental mechanisms regulate venation patterns, and therefore physiological performance, in monocot leaves.

## Author contributions

CP and ST contributed equally to this work.
